# Linking Data in a Shared Service Environment

**DOI:** 10.23889/ijpds.v9i5.2548

**Published:** 2024-09-18

**Authors:** Lisa Mirel, Cordell Goldent, Rob Zybrick, Rui Wang, Chrystine Tadler, Christine Cox

**Affiliations:** 1 National Center for Science and Engineering Statistics, National Science Foundation; 2 National Center for Health Statistics, Centers for Disease Control and Prevention; 3 HealthVerity, Inc.; 4 Mathematica; 5 NORC

Many federal agencies in the U.S. have begun exploring the use of privacy enhancing technologies, such as privacy preserving record linkage (PPRL), to integrate data from disparate sources to support evidence building and high-quality research. Recent legislation, including the Foundations for Evidence-Based Policymaking Act of 2018 and the CHIPS and Science Act of 2022, and recommendations from the Advisory Committee on Data for Evidence Building support the establishment of a shared service environment, namely the National Secure Data Service (NSDS), to facilitate this work. This talk will provide an overview of two NSDS Demonstration projects that utilize PPRL to integrate data covered by different legal provisions. The first project aims to link data between two federal statistical agencies and the objective of the second is to link data from a federal statistical agency and its parent agency. The talk will focus on the development of the required data sharing agreements, legal provisions that needed to be considered, security and privacy measures that had to be addressed, the software and IT infrastructure required, and the distinct PPRL tools utilized for each project. The talk will conclude with a presentation of preliminary linkage results, lessons learned, and a summary of how these projects will inform potential future data sharing efforts between and within agencies through the establishment of a future NSDS.

